# Case Report: Segment-specific minimally invasive surgical management of lumbar spinal epidural lipomatosis with adjacent-level degenerative stenosis

**DOI:** 10.3389/fsurg.2026.1876422

**Published:** 2026-08-19

**Authors:** Tongtong Zhang, Fuhai Yan, Lingna Shi, Hui Zhang

**Affiliations:** 1 Department of Spine Surgery, Gansu Provincial Hospital, Lanzhou, China; 2 The First Clinical Medical College of Gansu University of Chinese Medicine, Lanzhou, China; 3 Department of Pediatrics, Gansu Provincial Hospital, Lanzhou, China

**Keywords:** lumbar vertebrae, minimally invasive surgical procedures, spinal epidural lipomatosis, spinal fusion, spinal stenosis, spondylolisthesis

## Abstract

**Background:**

Spinal epidural lipomatosis (SEL) is characterized by excessive accumulation of unencapsulated adipose tissue within the epidural space and may cause clinically relevant neural compression. When SEL coexists with degenerative stenosis at an adjacent lumbar level, the involved segments may require different decompression and stabilization strategies.

**Case presentation:**

A 56-year-old man presented with chronic low back pain, progressive bilateral lower-extremity pain and numbness, and neurogenic claudication. Lumbar magnetic resonance imaging demonstrated excessive posterior epidural adipose tissue at L3-L4 with marked dural sac compression, together with degenerative central and lateral recess stenosis and Meyerding grade I degenerative spondylolisthesis at L4-L5. A single-stage, segment-specific minimally invasive procedure was performed. Tubular unilateral laminotomy with bilateral decompression and debulking of epidural adipose tissue were performed at L3-L4, whereas decompression, transforaminal lumbar interbody fusion, and bilateral pedicle screw fixation were performed at L4-L5. The operative time was 150 min, and estimated blood loss was 100 mL.

**Outcome:**

Early postoperative axial CT demonstrated the L3-L4 laminotomy and enlargement of the osseous canal. Side-by-side sagittal T2-weighted images obtained in comparable planes showed reduced posterior indentation of the dural sac at L3-L4 after surgery. At the 12-month follow-up, comparison with the early postoperative radiographs showed no obvious interval hardware displacement or loss of L4-L5 alignment. Plain radiographs did not establish solid interbody fusion.

**Conclusion:**

This case illustrates a segment-specific minimally invasive strategy for adjacent lumbar pathologies with different treatment requirements. Decompression without fusion may preserve an otherwise stable SEL-involved segment, whereas fusion at an adjacent degenerative level should be based on patient-specific clinical, radiological, and biomechanical considerations. Comparative safety or effectiveness cannot be inferred from a single case.

## Introduction

Spinal epidural lipomatosis (SEL) is characterized by excessive accumulation of unencapsulated adipose tissue within the epidural space, reducing the available spinal canal area and, in symptomatic cases, compressing the dural sac or nerve roots (1). Magnetic resonance imaging (MRI) is the principal diagnostic modality because epidural adipose tissue demonstrates signal characteristics similar to subcutaneous fat. Advanced lumbar SEL may produce stellate or Y-shaped deformation of the dural sac. The Borré classification provides an MRI-based framework for grading lumbosacral epidural fat overgrowth according to the relative dimensions of epidural fat, the dural sac, and the spinal canal (2).

SEL has been associated with chronic exogenous glucocorticoid exposure, obesity, endogenous hypercortisolism, and metabolic abnormalities, although no identifiable secondary cause is found in some patients (1, 3). Conservative management may include treatment of an underlying endocrine disorder, reduction or withdrawal of exogenous glucocorticoids when clinically appropriate, and weight reduction. Surgical decompression is generally considered when clinically relevant neural compression persists despite nonoperative treatment or when neurological symptoms progress (1, 4).

Surgical planning becomes more complex when SEL coexists with degenerative stenosis at an adjacent level because the pathological and biomechanical characteristics of the involved segments may differ. Decompression with preservation of the posterior stabilizing structures may be sufficient at an SEL-dominant level without spondylolisthesis, whereas the indication for fusion at an adjacent degenerative level requires individualized assessment. This report describes segment-specific minimally invasive treatment of symptomatic L3-L4 SEL and adjacent L4-L5 degenerative stenosis with grade I spondylolisthesis. The educational focus is the clinical-radiological correlation and the rationale for decompression without fusion at L3-L4 while adding interbody fusion and fixation at L4-L5.

## Case description

### Patient information

A 56-year-old man presented with a 1-year history of low back pain that had progressively worsened during the preceding 6 months and was accompanied by bilateral lower-extremity pain, numbness, and intermittent neurogenic claudication. Symptoms were aggravated by standing and walking and were partially relieved by rest. Nonoperative treatment, including nonsteroidal anti-inflammatory medication, had not provided clinically meaningful relief. No history of chronic systemic glucocorticoid therapy, repeated epidural steroid injections, known Cushing syndrome, hypothyroidism, or previous spinal surgery was documented. His height was 170 cm, weight was 76 kg, and body mass index was 26.3 kg/m^2^. Because the patient was overweight and a detailed metabolic assessment was unavailable for this retrospective report, the SEL was not categorically classified as idiopathic.

### Clinical findings

Tenderness and percussion tenderness were present over the lumbar region, with mildly restricted lumbar motion. Sensory examination demonstrated hypoesthesia over the anterior and posterolateral aspects of the left thigh, the lateral aspect of the left lower leg, and the posterior aspect of the right thigh. Lower-extremity motor strength and tendon reflexes were preserved bilaterally. Straight-leg-raising and reinforcement tests were negative, and no pathological reflexes were elicited.

### Diagnostic assessment

**Figure 1:**
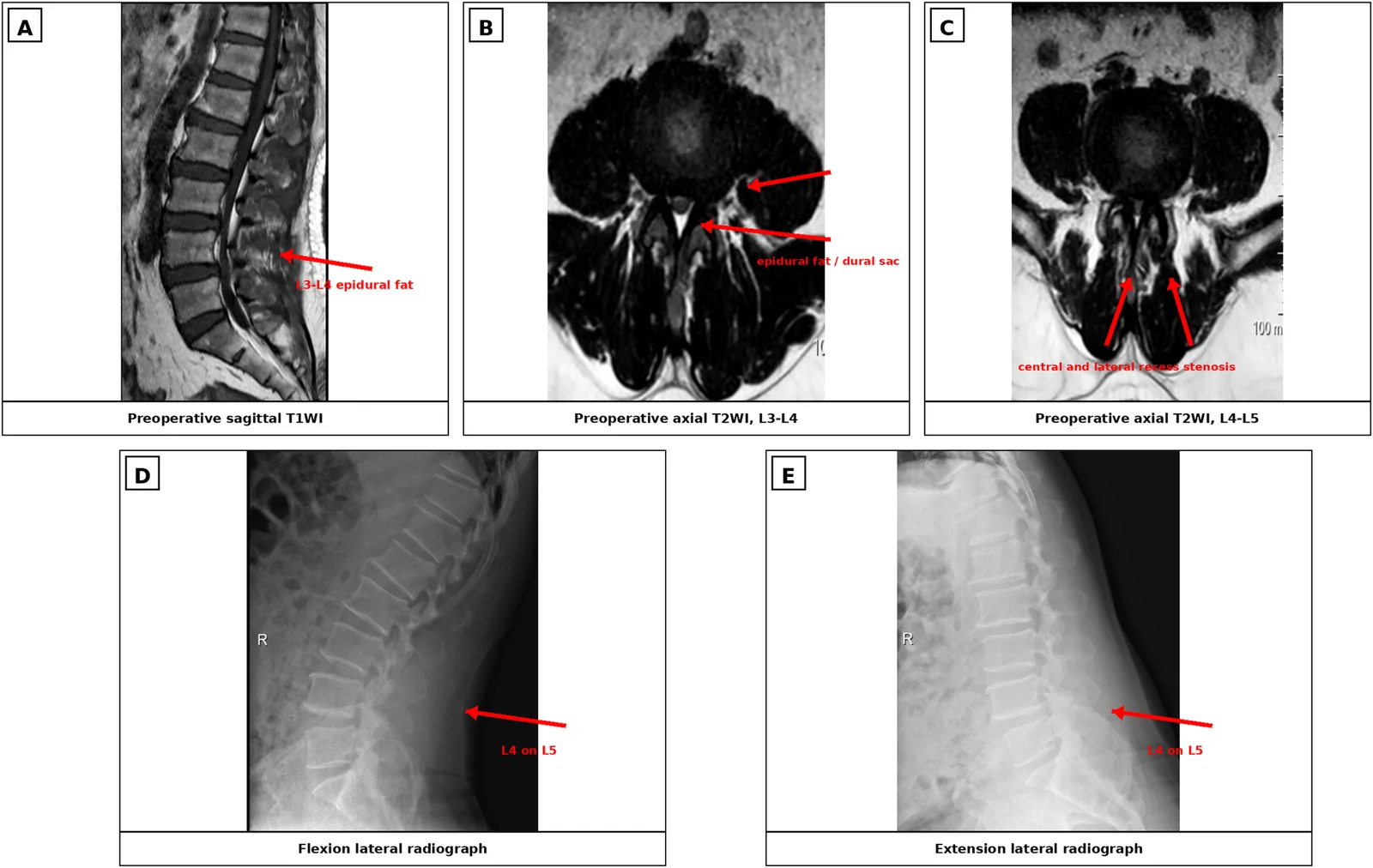
Preoperative diagnostic imaging. **(A)** Preoperative sagittal T1-weighted MRI showing high-signal posterior epidural adipose tissue at L3-L4 (arrow). **(B)** Preoperative axial T2-weighted MRI at L3-L4 showing excessive posterior epidural adipose tissue and deformation of the dural sac (arrows). **(C)** Preoperative axial T2-weighted MRI at L4-L5 showing degenerative central canal and bilateral lateral recess stenosis (arrows). **(D)** Flexion and **(E)** extension lateral radiographs showing Meyerding grade I anterior spondylolisthesis of L4 on L5 (arrows). Each panel directly identifies the sequence, anatomical level, and position.

Preoperative lumbar MRI included sagittal T1-weighted, sagittal T2-weighted, and axial T2-weighted sequences; an axial T1-weighted sequence was not acquired. Figure 1 presents the preoperative diagnostic images in a single composite figure: sagittal T1-weighted MRI showing posterior epidural fat at L3-L4, axial T2-weighted MRI at L3-L4 showing epidural fat-related dural sac deformation, axial T2-weighted MRI at L4-L5 showing degenerative central and bilateral lateral recess stenosis, and flexion and extension radiographs labeled separately. Standing flexion-extension radiographs showed Meyerding grade I anterior spondylolisthesis of L4 on L5. No pars interarticularis defect was identified, supporting a degenerative rather than isthmic etiology. Because quantitative translational and angular motion had not been recorded, the lesion is described as degenerative spondylolisthesis rather than objectively proven dynamic instability. Formal Borré grading and quantitative canal-area measurements had not been performed during the initial clinical evaluation and could not be reconstructed reliably from the available exported images.

Although the L4-L5 stenosis and spondylolisthesis could account for the neurogenic claudication and a substantial proportion of the lower-extremity symptoms, the L3-L4 SEL was not considered an incidental finding. The epidural adipose tissue produced marked level-specific dural sac compression, and the anterior-thigh sensory disturbance was compatible with upper lumbar neural involvement. The lateral lower-leg symptoms and claudication were considered more consistent with L4-L5 stenosis. Because symptom distributions in multilevel lumbar disease frequently overlap, the decision to decompress both levels was based on the combined neurological and radiological findings rather than on a single dermatome.

### Therapeutic intervention

After induction of general anesthesia, the patient was placed prone, and the operative levels were confirmed fluoroscopically. A left-sided paramedian working corridor was selected because the left-sided sensory symptoms were more extensive and because this was the surgeon's preferred side for the transforaminal interbody procedure. Although symptoms were bilateral, the tubular retractor permitted contralateral decompression using an over-the-top technique.

At L4-L5, the ipsilateral and contralateral lateral recesses were decompressed through an expandable tubular retractor. Discectomy, endplate preparation, interbody grafting and cage placement, and bilateral pedicle screw fixation were then completed. Fusion was selected because of persistent mechanical low back pain, degenerative spondylolisthesis, stenosis requiring bilateral decompression, and concern that the extent of decompression required at this level could further compromise segmental stability. Grade I degenerative spondylolisthesis alone was not regarded as an absolute indication for fusion.

The tubular working corridor was subsequently redirected cranially to L3-L4. Partial laminotomy, resection of the ligamentum flavum, and careful piecemeal debulking of abundant yellow epidural adipose tissue were performed. Contralateral decompression was achieved using an over-the-top technique while preserving most of the facet joints and posterior ligamentous structures. The dural sac expanded after decompression. Fusion was not extended to L3-L4 because no spondylolisthesis or pars defect was present at that level and the facet-preserving unilateral laminotomy with bilateral decompression technique was intended to limit the risk of iatrogenic instability. The total operative time was 150 min, and estimated blood loss was 100 mL.

### Postoperative course and follow-up

**Figure 2:**
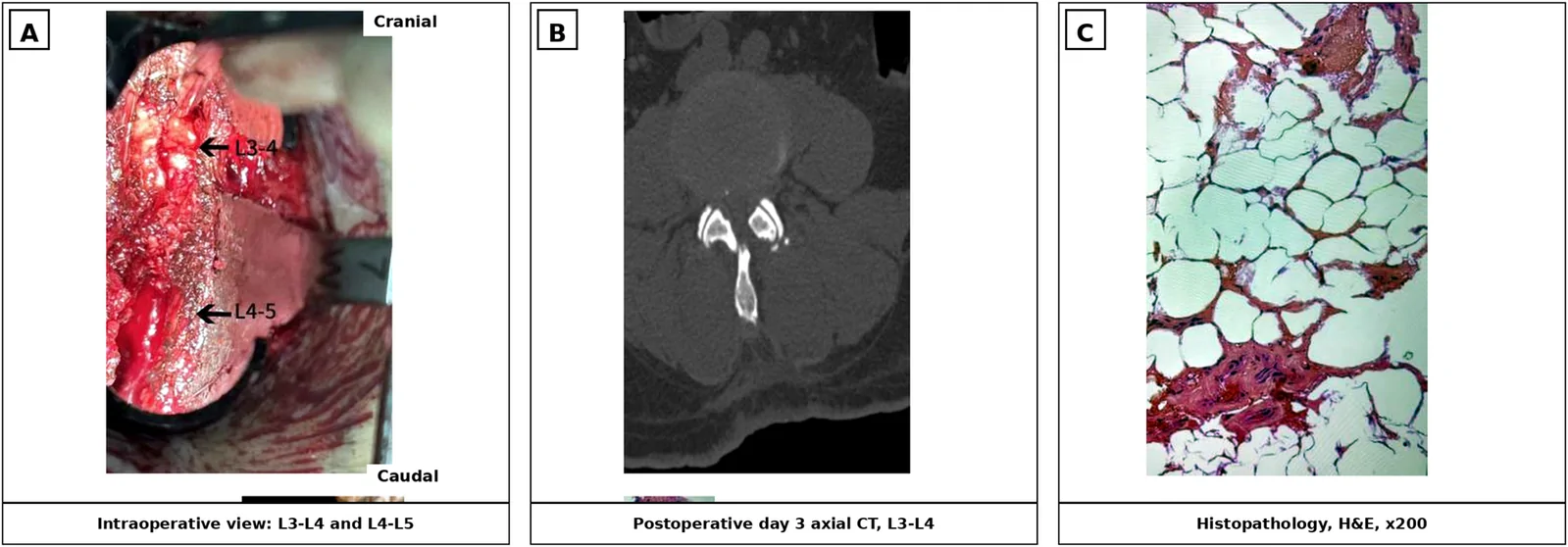
Intraoperative, early postoperative CT, and histopathological findings. **(A)** Intraoperative view showing the L3-L4 and L4-L5 operative levels, with cranial and caudal orientation indicated. **(B)** Axial CT obtained on postoperative day 3 at L3-L4 showing the laminotomy and enlargement of the osseous spinal canal. **(C)** Mature adipose tissue admixed with fibrous and vascular components (hematoxylin and eosin staining, original magnification x200).

Postoperatively, the patient received analgesia and routine perioperative care. Drainage tubes were removed on postoperative day 2, and ambulation with a lumbar brace was permitted. The intraoperative view, postoperative day 3 axial CT, and histopathological findings are presented together in Figure 2 according to their clinical purpose. The CT image demonstrates the L3-L4 laminotomy and enlargement of the osseous canal; it is not used to make a quantitative claim of complete decompression. Histopathological examination demonstrated mature adipose tissue admixed with fibrous tissue and vascular components, consistent with the resected epidural adipose tissue.

**Figure 3:**
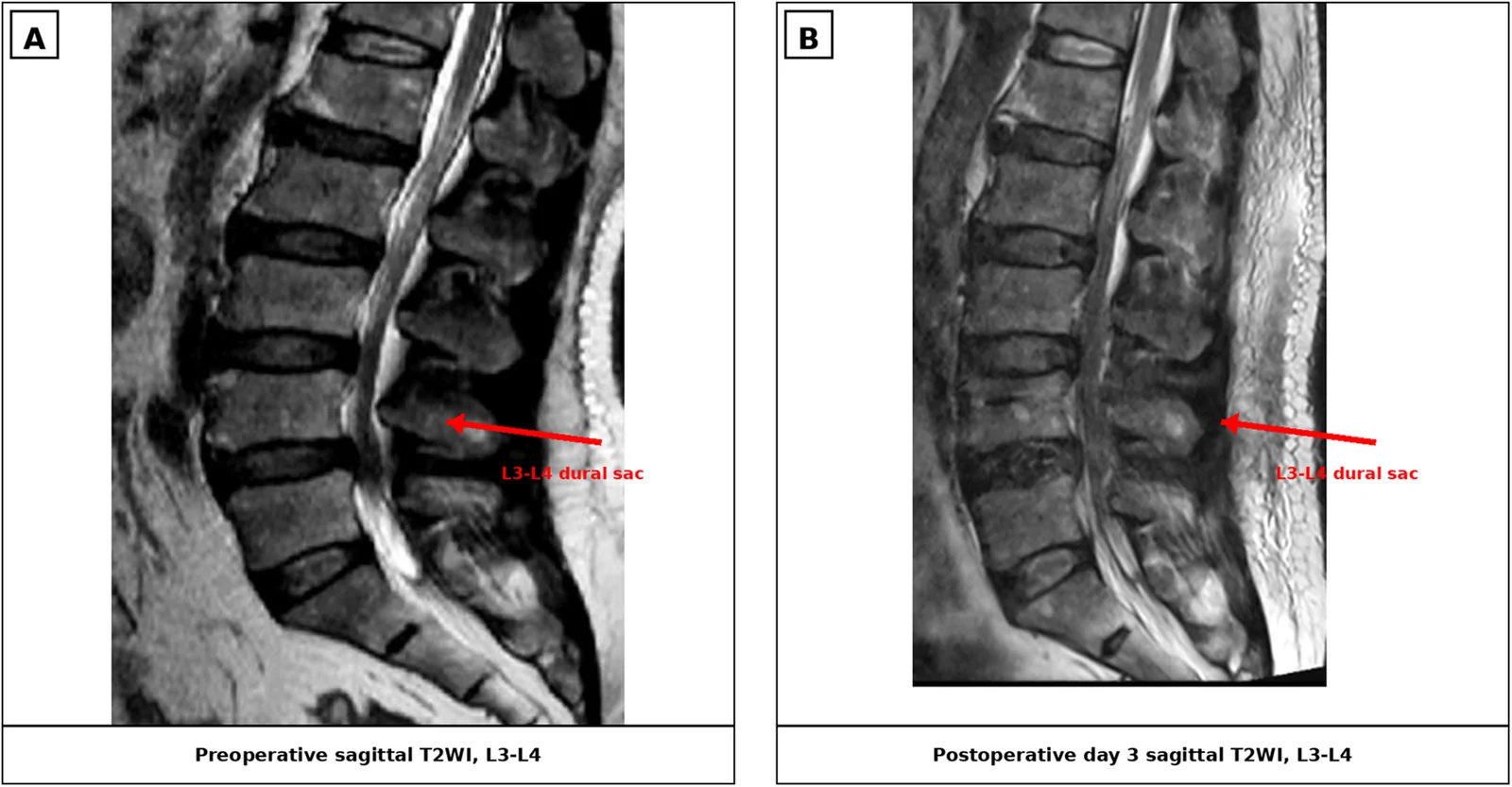
Direct preoperative and postoperative sagittal T2-weighted MRI comparison at L3-L4. **(A)** Preoperative sagittal T2-weighted MRI showing posterior indentation and compression of the dural sac at L3-L4. **(B)** Sagittal T2-weighted MRI obtained on postoperative day 3 in the closest comparable anatomical plane, showing reduced posterior indentation of the dural sac and increased visible cerebrospinal fluid space at L3-L4. The level and relevant dural sac region are marked consistently in both panels.

Preoperative and postoperative day 3 sagittal T2-weighted images obtained in the closest comparable anatomical planes are presented side by side in Figure 3. Compared with the preoperative image, the postoperative image shows reduced posterior indentation of the dural sac and increased visible cerebrospinal fluid space at L3-L4. Because the examinations were acquired for routine clinical care, the images are comparable but not identical slices.

**Figure 4:**
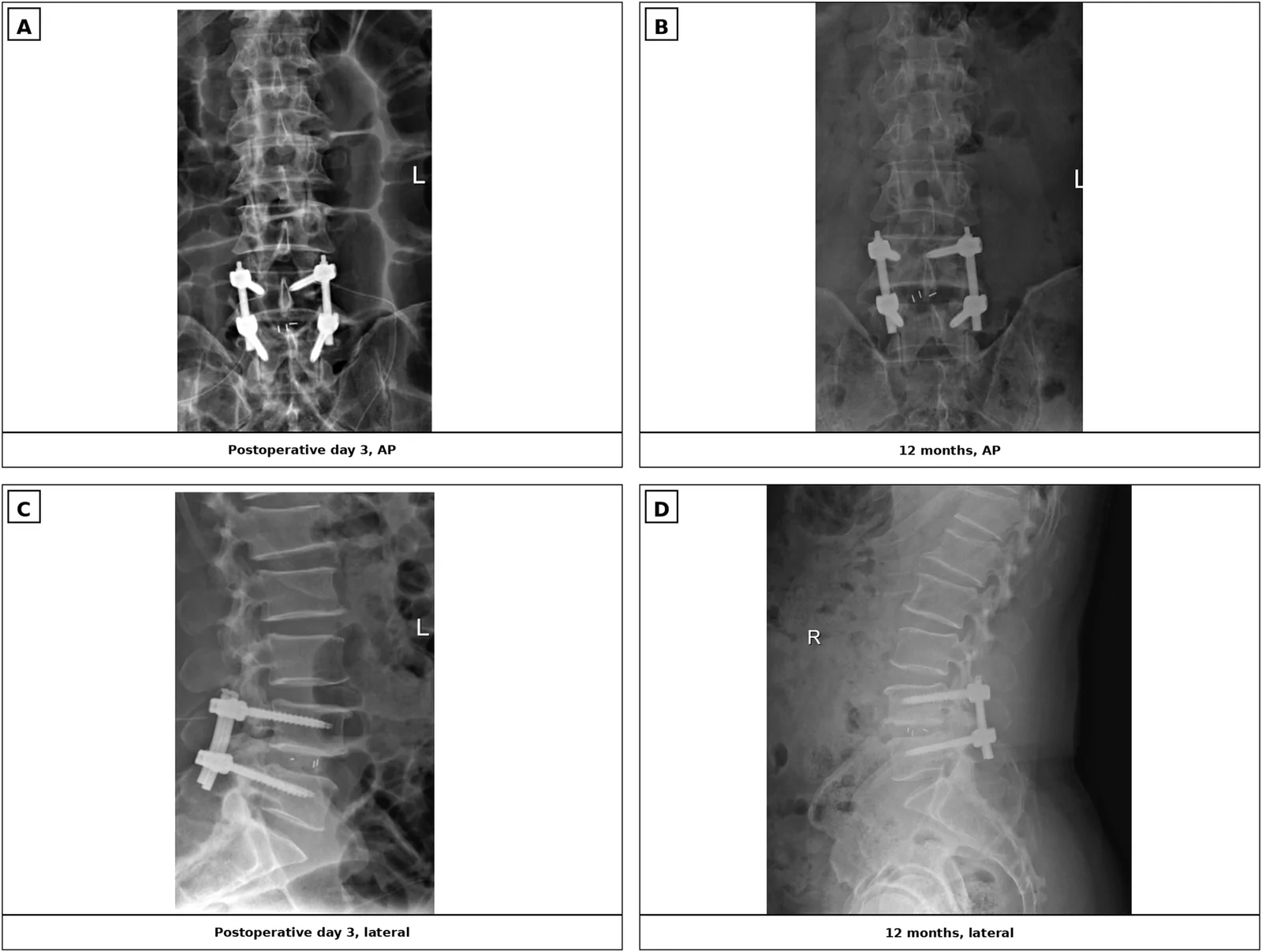
Early postoperative and one-year radiographic comparison. **(A)** Anteroposterior radiograph obtained on postoperative day 3. **(B)** Anteroposterior radiograph obtained at 12 months. **(C)** Lateral radiograph obtained on postoperative day 3. **(D)** Lateral radiograph obtained at 12 months. No obvious interval hardware displacement, screw or rod breakage, cage migration, or loss of L4-L5 alignment was observed. Plain radiographs alone did not establish solid interbody fusion.

At the 12-month follow-up, the patient reported sustained relief of low back pain and lower-extremity pain, partial improvement in numbness, and improved walking tolerance. He remained independent in daily activities and had not required reoperation; return-to-work status was not documented. Figure 4 directly compares the early postoperative and 12-month anteroposterior and lateral radiographs. No obvious interval displacement of the pedicle screws, rods, or cage, hardware breakage, cage migration, or loss of L4-L5 alignment was observed. Plain radiographs were insufficient to confirm solid interbody fusion, and no follow-up CT was obtained. No follow-up MRI was available to formally assess recurrent epidural fat-related compression. Standardized visual analogue scale and Oswestry Disability Index scores had not been prospectively collected, limiting quantitative outcome assessment.

### Timeline

The clinical course is summarized in Supplementary Table S1 in accordance with the CARE reporting framework.

## Discussion

The principal educational value of this case is the application of different operative objectives to adjacent segments with distinct pathological characteristics. At L3-L4, the dominant lesion was epidural adipose tissue causing direct dural sac compression. At L4-L5, the pathology consisted of degenerative osseoligamentous stenosis associated with grade I degenerative spondylolisthesis. The operative plan was therefore formulated separately for each level rather than extending fusion routinely across both segments.

Clinical-radiological correlation was essential because L4-L5 stenosis alone could plausibly explain the claudication and much of the lower-extremity symptom burden. In this patient, however, the L3-L4 epidural fat produced substantial, level-specific dural sac compression and was accompanied by anterior-thigh sensory disturbance compatible with upper lumbar neural involvement. These findings supported treatment of both levels, while acknowledging that dermatomal symptoms frequently overlap in multilevel lumbar degeneration. Observational outcome studies indicate that surgical decompression can improve pain and walking capacity in symptomatic SEL (4).

At L3-L4, decompression without fusion was selected because no spondylolisthesis or pars defect was present and because tubular unilateral laminotomy with bilateral decompression preserved most of the facet joints, spinous process, and contralateral musculoligamentous structures. Any osseous decompression may contribute to iatrogenic instability if the facets and posterior tension band are excessively disrupted; however, unilateral laminotomy with bilateral decompression is intended to reduce this structural disruption and has shown favorable outcomes in lumbar stenosis cohorts (6). Han et al. described posterior decompression and fusion for obesity-associated SEL combined with lumbar degenerative stenosis (5). In contrast, the present strategy limited fusion to the adjacent L4-L5 level because L3-L4 lacked spondylolisthesis and was treated with a facet-preserving decompression. Extending instrumentation to L3-L4 would have increased the fusion length and exposed an otherwise stable segment to unnecessary motion loss and adjacent-level loading.

The indication for fusion at L4-L5 warrants balanced interpretation. Randomized evidence indicates that decompression alone can provide outcomes noninferior to decompression with instrumented fusion in many patients with low-grade degenerative spondylolisthesis, including at 5-year follow-up (7, 8). In contrast, the smaller SLIP trial suggested that selected patients with stable grade I degenerative spondylolisthesis may derive some benefit from fusion, including a lower reoperation rate (9). Accordingly, grade I spondylolisthesis alone was not considered sufficient justification for TLIF. In the present patient, fusion was selected because of persistent mechanical low back pain, degenerative spondylolisthesis, the need for bilateral decompression, and concern that the required decompression could further compromise segmental stability. This explanation reflects individualized surgical judgment rather than a claim that fusion is mandatory for all comparable patients.

A single-stage procedure was selected because both lesions were considered clinically relevant and could be treated during the same anesthetic session through adjacent minimally invasive working corridors. The left-sided approach was chosen on the basis of the more extensive left-sided sensory symptoms and surgeon preference; the over-the-top technique allowed bilateral decompression despite bilateral symptoms. Compared with open TLIF, MIS-TLIF is associated with less perioperative tissue disruption and blood loss, while long-term patient-reported outcomes are generally comparable (10). These general advantages do not establish superiority of the present strategy over decompression alone, open surgery, or staged treatment.

This report has several limitations. First, it represents a single patient and cannot establish comparative effectiveness, safety, or superiority. Second, the MRI scanner field strength and complete acquisition parameters were unavailable in the retrospective record; an axial T1-weighted sequence was not acquired, and formal Borré grading and canal-area measurements were not available. Third, quantitative flexion-extension measurements were not recorded; therefore, the manuscript does not describe objectively proven dynamic instability. Fourth, the preoperative and postoperative sagittal T2-weighted images were selected from the closest comparable planes but were not identical slices. Fifth, standardized visual analogue scale and Oswestry Disability Index scores were not prospectively collected. Sixth, radiological follow-up at 12 months relied on standing plain radiographs: CT confirmation of solid fusion and MRI assessment of recurrent epidural fat were unavailable. Finally, the exact contribution of each level to the overlapping bilateral symptoms cannot be determined with certainty. Longer follow-up with standardized patient-reported outcomes, dynamic radiographs, CT-based fusion assessment, and MRI surveillance would strengthen the conclusions.

## Conclusion

This case illustrates a segment-specific minimally invasive strategy for symptomatic L3-L4 spinal epidural lipomatosis with adjacent L4-L5 degenerative stenosis and spondylolisthesis. Decompression without fusion was used at the SEL-involved level to preserve an otherwise stable segment, whereas TLIF was performed at the adjacent degenerative level on the basis of individualized clinical, radiological, and biomechanical considerations. This approach may be considered in carefully selected patients, but its comparative safety and effectiveness cannot be established from a single case.

## Data Availability

The raw data supporting the conclusions of this article will be made available by the authors, without undue reservation.

## Patient perspective

At the 12-month follow-up, the patient reported sustained relief of low back pain and lower-extremity symptoms, improved walking tolerance, and independence in daily activities. He expressed satisfaction with the postoperative recovery.
