# Supplementary material for: Case Report: Segment-specific minimally invasive surgical management of lumbar spinal epidural lipomatosis with adjacent-level degenerative stenosis
Source: Front Surg. 2026 Aug 19;13:1876422. doi: 10.3389/fsurg.2026.1876422 (PMC13533961; doi:10.3389/fsurg.2026.1876422)
Supplement: Supplementary file 1 [file Table1.docx]

Supplementary Table S1. Clinical Timeline

| Time point | Clinical events |
| --- | --- |
| 1 year before admission | Onset of low back pain without a clear precipitating event. |
| 6 months before admission | Progressive low back pain with bilateral lower-extremity pain, numbness, and neurogenic claudication. |
| Before admission | Nonoperative treatment, including nonsteroidal anti-inflammatory medication, failed to provide meaningful relief. |
| At admission | Bilateral lower-extremity sensory disturbance without motor weakness or pathological reflexes. |
| Preoperative imaging | L3-L4 SEL with marked dural sac compression and L4-L5 degenerative stenosis with Meyerding grade I degenerative spondylolisthesis. |
| Surgery | Single-stage L3-L4 tubular unilateral laminotomy with bilateral decompression and epidural adipose tissue debulking, together with L4-L5 decompression, TLIF, and bilateral pedicle screw fixation. Operative time, 150 min; estimated blood loss, 100 mL. |
| Postoperative days 2-3 | Drain removal and mobilization with a brace. Early CT and MRI documented the postoperative anatomical findings; early radiographs documented implant position. |
| 12-month follow-up | Sustained symptomatic and walking-tolerance improvement without reoperation. Comparison with early postoperative radiographs showed no obvious interval hardware displacement or loss of L4-L5 alignment. Plain radiographs could not confirm solid fusion; follow-up CT, MRI, and standardized outcome scores were unavailable. |
